# Visualising Geopolymerisation Processes Using Scanning X-Ray Diffraction and Fluorescence Microscopy

**DOI:** 10.3390/ma17235896

**Published:** 2024-12-02

**Authors:** Grant A. van Riessen, Gerard N. Hinsley, Cameron M. Kewish, Arie van Riessen

**Affiliations:** 1 Department of Mathematical and Physical Sciences, School of Computing, Engineering and Mathematical Sciences, La Trobe University, Bundoora, VIC 3086, Australia; 2 Deutsches Elektronen-Synchrotron DESY, Notkestrasse 85, D-22607 Hamburg, Germany; 3 Australian Nuclear Science and Technology Organisation, Australian Synchrotron, Clayton, VIC 3168, Australia; 4 John de Laeter Centre, Curtin University Australia, Perth, WA 6845, Australia

**Keywords:** geopolymer, metakaolin, in situ microscopy

## Abstract

In situ observation of the dissolution of metakaolin followed by the condensation of geopolymer was performed by a combination of synchrotron X-ray fluorescence microscopy and scanning X-ray diffraction microscopy. New insight into the complex geopolymerisation process was obtained by simultaneously acquiring compositional and morphological information. The combination of selected alkali and experimental conditions produced a geopolymer with the targeted composition but resulted in the complete and rapid dissolution of metakaolin followed by immediate geopolymer formation. The geopolymer microstructure continued to evolve, along with pore growth, over several hours.

## 1. Introduction

Alkali-activated aluminosilicate inorganic polymers or geopolymers have been the focus of many investigations, for applications varying from Portland cement replacement [1], radioactive waste containment [2], fireproof barriers [3], and ceramic precursors [4]. Despite the recent proliferation of reliable geopolymer products, the fundamental structure of geopolymers and their formation mechanisms are not yet completely understood. An increased understanding of the formation of geopolymers will lead to the development of products with superior properties, especially durability.

In model systems of clay-based geopolymers the basic reaction mechanisms can be described as a dissolution–reorientation–solidification process [5]. The dissolution of the solid precursor, such as metakaolin, starts immediately upon contact with an alkali–silicate solution. The polycondensation of silicon and aluminium tetrahedra species subsequently occurs. The physicochemical properties of the resulting geopolymer including strength, porosity, permeability and composition depends primarily on water content and the specific ratio of Si:Al and Al:M, where M is the cation in the alkali–silicate solution [6,7].

The relationship between geopolymer composition and microstructure has generally been studied indirectly and, typically after the completion of the geopolymerisation process. The presence of unreacted particles in hardened geopolymer indicates incomplete precursor dissolution [8,9], leading to Si:Al ratios that differ from target values.

Direct, in situ study is complicated by the combination of high alkali concentrations, high ratios of solids to liquids, and the dynamic relationship between heterogeneous structure and chemistry. Consequently, mechanistic studies of geopolymerisation have generally been limited by the inability to simultaneously monitor compositional and morphological changes at relevant length and time scales. In particular, previous studies of geopolymerisation using synchrotron radiation have been limited by either poor temporal or spatial resolution [10,11,12,13,14]. The former is a particular problem for the early stages of the reaction when most of the dissolution and polycondensation reactions occur [15,16]. The latter results in bulk-averaged information that obscures the significance of the local microstructure and heterogeneity of the composition [17,18]. A critical aspect of this research is to establish at what point the formation of the geopolymer gel at the alkali/aluminosilicate interface prevents further dissolution of the aluminosilicate and prematurely fixes a sub-optimal Si:Al ratio.

The feasibility of rapid data acquisition using on-the-fly scanning with a fast Eiger detector has been demonstrated at the X-ray fluorescence microscopy (XFM) beamline at the Australian Synchrotron [19,20]. In a proof-of-principle experiment, we previously demonstrated the ability to follow the progress of geopolymerisation at the interface between a metakaolin-based geopolymer paste and alkali solution. Scanning X-ray diffraction microscopy (SXDM) phase images with spatial resolution < 100 nm were obtained simultaneously with scanning X-ray fluorescence microscopy (SXFM) images at 10 min intervals over 16 h [13]. Systematic morphological changes on the sub-micrometre length scale could be recognised.

The XFM beam configuration was subsequently optimised for SXDM with a ∼500 nm focus and high coherence, considerably improving the reliability of SXDM image reconstruction compared to that in our previous work [13,21]. Here, we describe a simple approach for the in situ observation of the geopolymerisation process using simultaneous SXDM and SXFM. A metakaolin-based geopolymer and caesium silicate activator is chosen to avoid the complexity that arises from more compositionally heterogeneous aluminosilicate precursors and for the relative simplicity of the resulting microstructure [6]. We show that the unreacted metakaolin precursor is distinguishable from aluminosilicate gel by SXDM. The dynamic evolution of the aluminosilicate gel microstructure is observed in situ by SXDM, while the redistribution of the elements contained in the silicate precursors can be followed by SXFM.

## 2. Samples

### 2.1. Sample Presentation

Mixing metakaolin (anhydrous, calcined form of the clay mineral kaolinite) and caesium silicate in a ratio typical for geopolymer production produces a viscous slurry that can be difficult to prepare in a way that satisfies sample thickness constraints for two-dimensional SXDM imaging. Sandwiching a small volume between two Si_3_N_4_ membranes was found to provide films of appropriate thickness, but X-ray-beam-induced radiolysis [22] produced bubbles which, trapped between the membranes, displaced the metakaolin/geopolymer material and prevented the tracking of the geopolymerisation process. To circumvent this problem, the slurry was simply drop-deposited onto a Si_3_N_4_ window, leaving it exposed to air. The thickness of the deposit was reduced by increasing the wettability of the Si_3_N_4_ surface by prior plasma treatment and by adding water to the slurry before deposition. Excess water is expected to increase pore size [23] and slow the dissolution process [24]. However, after drop deposition, water is rapidly lost by evaporation due to the high surface-to-volume ratio, driving the composition of the alkali activator back towards the target value. While it was not possible to quantify the rate of water loss in situ, the validity of the method was evaluated based on the composition of the cured geopolymer (SEM/EDS section).

### 2.2. Sample Preparation

Caesium silicate solution was prepared by dissolving silica fume (Doral SF98, Rockingham, Australia) in caesium hydroxide (Sigma Aldrich, Castle Hill, Australia; 99.9% purity). Metakaolin (Metamax, BASF Corporation, Florham Park, NJ, USA) and caesium silicate were mixed to make the geopolymer. The metakaolin used in this study has an average particle size of 1.3 μm. The target formulation was Cs_2_O·Al_2_O_3_·4SiO_2_·11H_2_O [25], to which extra water was added until the viscosity was sufficiently low so that a drop deposited on a plasma-treated Si_3_N_4_ membrane would spontaneously spread to form a film of less than 5 μm in thickness. The samples were immediately transferred to the XFM beamline for analysis.

One sample was prepared from a metakaolin/caesium silicate mixture that was stored in a sealed container for 12 h and rinsed with acetone, displacing water and effectively terminating the reaction.

Samples of metakaolin only were also prepared by drop-depositing a suspension in ethanol onto a Si_3_N_4_ membrane and allowing it to dry.

## 3. In Situ X-Ray Imaging

Experiments were performed at the XFM beamline at the Australian Synchrotron [26]. Scanning X-ray diffraction microscopy was recently developed at this facility and can be performed simultaneously with scanning fluorescence microscopy using a continuous fly scanning scheme that has previously been described [20]. An 8 keV X-ray beam was conditioned by aperturing to provide high spatial coherence and focussed to a sub-micron spot at the sample plane using a Kirkpatrick–Baez mirror pair.

**Figure 1:**
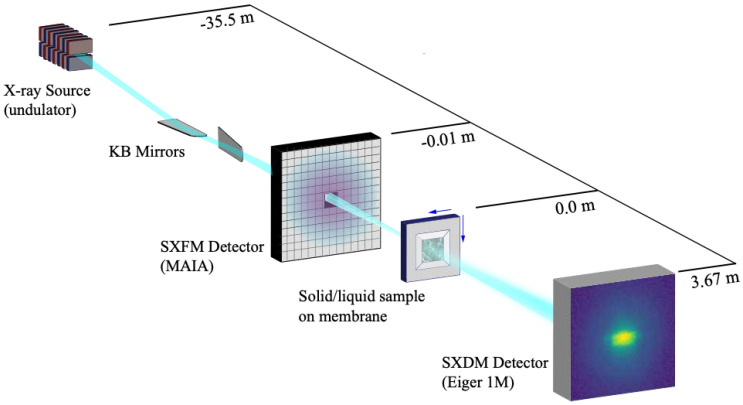
Simplified representation of experimental geometry (not to scale). The sample is scanned in the focal plane of the KB mirrors while X-ray fluorescence is detected in back-scatter geometry with the MAIA detector and coherent diffraction intensity is measured in the far field of the sample using the Eiger detector.

Low-resolution, coarse scans generating XFM and X-ray transmission maps were first performed to locate the sample at the beam focus and to select an area for further investigation. The samples were then fly-scanned through the focus over a 20 μm × 20 μm area using a bidirectional parabolic raster scanning trajectory to minimise overheads, where the horizontal axis (*x*-axis) acts as the fast axis. Using a 100 nm sampling interval in both the horizontal and vertical directions produced a set of 32,588 diffraction patterns for each SXDM dataset with an effective dwell time of 25 ms per diffraction pattern. The SXDM data (coherent diffraction patterns) were collected with an Eiger X 1M (Dectris, Baden-Daettwil, Switzerland) pixel-counting imaging detector with 75 × 75 μm pixels that was placed 3.67 m downstream of the focus. Fluorescence data were simultaneously collected with a 384-element MAIA (Revision C) detector [27] positioned 0.01 m upstream of the sample in backscatter geometry. Figure 1 illustrates the sample and detector geometry.

The imaging process described above was repeated to acquire data from the same region of each sample every 17 min over a period of many hours to follow changes resulting from the reaction of the precursors. Data were also collected from the reaction-terminated geopolymer sample and metakaolin sample using the same illumination and scan parameters.

Images were reconstructed from each set of diffraction patterns using the parallelised Python framework PtyPy [28] and using the Australian Synchrotron Computing Infrastructure (ASCI). A region of 256 × 256 pixels from each diffraction pattern was used, yielding an effective pixel size in the reconstructed images of 31 nm. The illumination function at the sample plane was simultaneously reconstructed independently with each of the sample images. Three coherent modes [29] for the probe were used in the reconstruction to account for the reduced coherence in the illumination that was introduced by fly-scanning. For each dataset, an initial estimate based on a simple model beam was used, and all of them converged to a similar result, confirming that the beam properties were stable over the duration of the experiment. The reconstructions were completed using 300 iterations of the difference map algorithm [30] and 200 iterations of the maximum-likelihood algorithm [31]. After the first 50 iterations, a probe position correction algorithm [32] implemented in PtyPy was applied.

The reconstructed phase images were corrected for phase wrapping artefacts and a linear-phase ramp artefacts. Due to the absence of any stable internal reference, the phase values in each reconstructed image were shifted such that the mean value was zero.

The SXFM spectra obtained at each scan interval were processed using GeoPIXE software (version 6.3) [33]. The energy scale was calibrated using spectra from reference standards and corrected for detector geometry and efficiency. Spectral features associated with characteristic X-ray emission from elements in the samples, Compton scattering, and elastic scattering were fitted to the spectrum acquired at each pixel, and maps of the distribution of Cs and contaminant metals were obtained. Further details are provided in Section 4.2.

## 4. Results

### 4.1. SXDM

**Figure 2:**
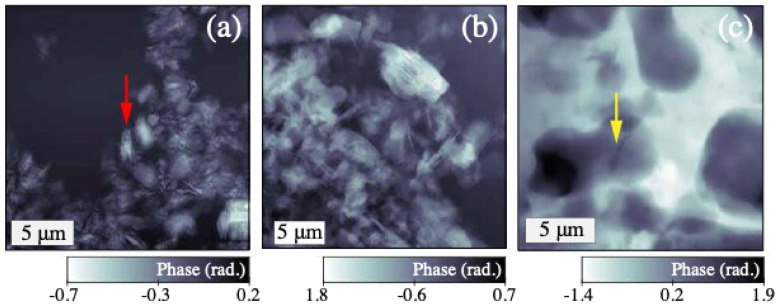
Representative phase images of precursor and reaction products. Pure metakaolin particles are shown (**a**), with a red arrow indicating the characteristic stacked arrangement of the metakaolin particles. Predominantly unreacted metakaolin can be recognised by its similar morphology in a metakaolin/caesium silicate mixture stored in a sealed container for 12 h until it was sampled and water was displaced to stop the reaction (**b**). A porous geopolymer network with no remaining metakaolin particles is observed in (**c**), a phase image obtained from sample 302 min after preparing and depositing a film of the metakaolin/caesium silicate mixture. In this case, the arrow indicates a nanoscale crack.

A representative SXDM phase contrast image obtained from the dry metakaolin sample without caesium silicate is shown in Figure 2a. Notably, the contrast appears to be dominated by the relative orientation of the metakaolin particles. The characteristic stacked arrangement of individual layers separated by 100–200 nm can be resolved, shown by a red arrow. The same distinctive layered metakaolin structure is seen in the metakaolin/caesium silicate 12 h reaction-terminated sample in Figure 2b. Amorphous geopolymer phases are not easily recognised in this region of interest. It appears that unreacted metakaolin was present when the reaction was terminated.

Comparison of the microstructure in the phase images in Figure 2 shows that SXDM provides the material sensitivity and spatial resolution required to identify the metakaolin precursor from its nanoscale morphology and to distinguish it from products of the polycondensation and geopolymerisation processes. A slurry deposited onto a silicon nitride membrane immediately after mixing and imaged after five hours exhibits a distinctly different microstructure consisting predominantly of a geopolymer with large (∼5 μm) pores (Figure 2c). While nanoscale structures are also resolved in this case, no unreacted metakaolin within the geopolymer matrix can be identified.

Complex images corresponding to phase images in Figure 2b,c were used to estimate the lower and upper bound to the sample-dependent resolution, which were 33 nm and 113 nm, respectively, from the ½-bit threshold of the Fourier ring correlation (FRC) [34]. FRC is widely used as the metric for determining the spatial resolution for super-resolution microscopic images [35]. The Fourier space correlation between two images is calculated for all spatial frequencies, therefore requiring two independent measurements to be taken on the same region. For the dynamic samples of this work, changes that occur between consecutively acquired images would reduce the correlation. A conservative estimate of the SXDM image resolution was instead obtained from the ½-bit threshold of the FRC computed from two complex images reconstructed from complimentary subsets of the same SXDM dataset.

**Figure 3:**
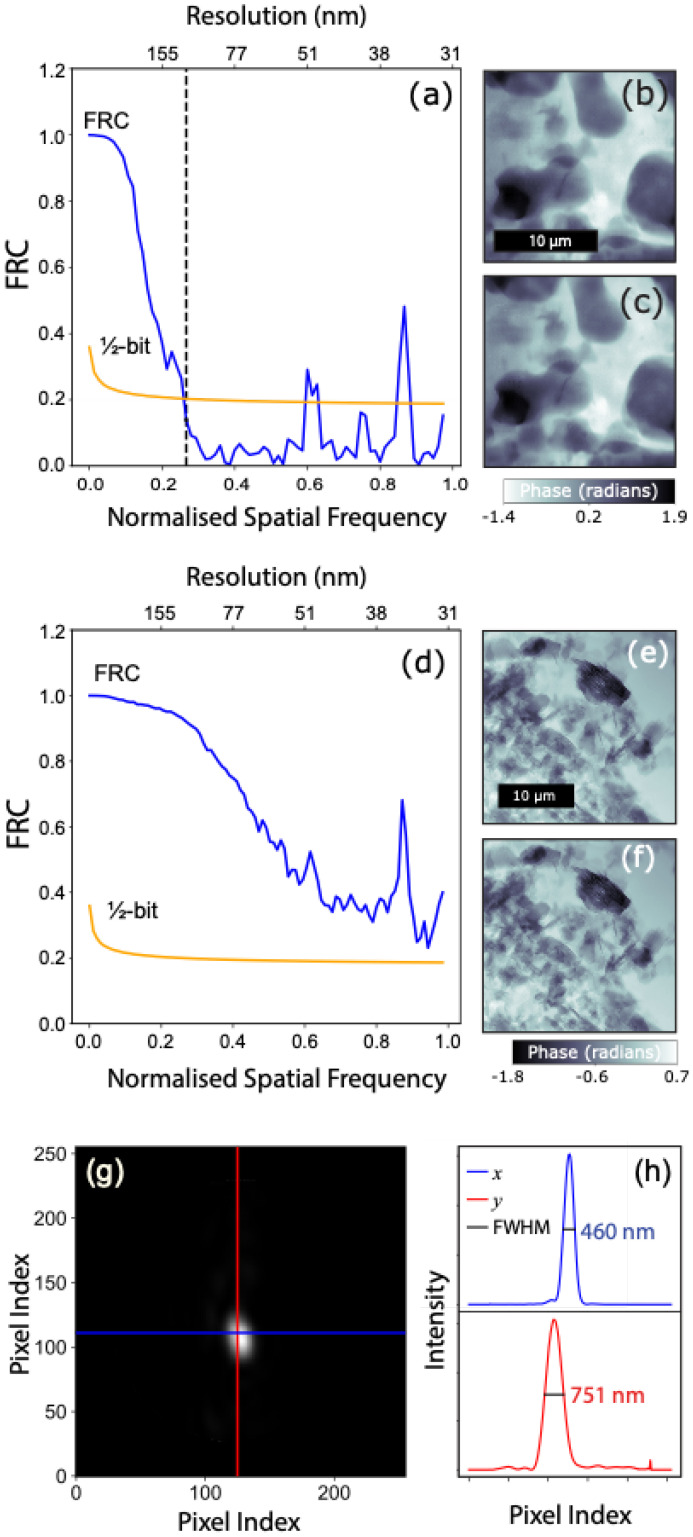
(**a**) SXDM spatial resolution estimated from the ½-bit threshold of the FRC, yielding an upper bound of 113 nm (dashed line), obtained from complex images (**b**,**c**). (**d**) The lower-bound SXDM resolution equivalent to the reconstructed object pixel size of 33 nm, obtained from complex images (**e**,**f**) reconstructed from data obtained from a stable sample consisting predominantly of unreacted metakaolin. (**g**) The intensity of a representative reconstructed probe used to estimate (**h**) the SXFM resolution from the beam FWHM was in the x and y directions.

By this approach, the resolution was estimated to be 113 nm for the last image in the time-series obtained from the metakaolin/caesium silicate sample (Section 4.3). While sufficient to distinguish components of the geopolymer paste and other microstructural characteristics, the ability to identify smaller features may be limited by the achievable contrast through thick samples. For this reason, we also examined images of the sample in which the reaction was terminated by displacing water with acetone and estimated the resolution to be equal to the reconstructed pixel size of 33 nm. Agglomerations of unreacted metakaolin particles were identified in the SXDM images obtained from this sample by comparison to the SXDM image of raw metakaolin (Figure 2). The two estimates, illustrated in Figure 3, represent the upper (113 nm) and lower (33 nm) bounds to the resolution that can be achieved under the imaging conditions that were employed in this work. The wide range between the upper and lower bounds is consistent with the expectation that spatial resolution in SXDM is strongly dependent on the object properties.

### 4.2. SXFM

Each detector element in the MAIA detector is used to record fluorescent photons as ‘events’, which contain information consisting of the detector channel, X-ray energy, and time-over-threshold [26]. These event data are continuously collected and delimited by pixel transits, which are defined by the translation of the sample scanning stage and scan geometry. The event data are paired with information of the pixel address, acquisition time interval, and integrated flux counts, then sorted into appropriate pixels to create an image [27]. This experiment consisted of an array of 200 × 200 pixel addresses, where the GeoPIXE software [33] was used to transform the event data, or integrated energy spectra, using the dynamic analysis (DA) method [36].

**Figure 4:**
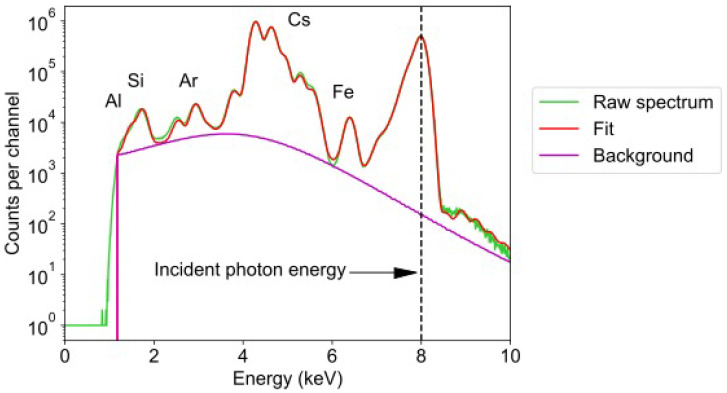
The raw energy spectrum of the fluorescence (green) collected by the MAIA detector for a single scan. This spectrum was fitted with the sum of the background and peaks representing fluorescence from the selected elements, and the elastic scatter peak (red). The selected elements for the fit were Al, Si, Ar, Fe, and Cs, where the L-lines of Cs were used. A vertical dashed black line indicates the incident photon energy of 8 keV.

The mean energy spectrum from all 384 detectors for the first time point is shown in Figure 4, along with the fitted sum of background and peaks representing the contribution of different elemental lines and the elastic scatter. The process of selecting elements to add into the fit can be aided by a priori knowledge of the expected chemical composition, as well as through complimentary techniques that can be performed post-experiment. The fluorescence spectrum here was fitted with the following selected elements, Al, Si, Ar, Fe, and Cs, where the L-lines of Cs were used due to the inability of 8 keV X-rays to excite the K-shell emission. Ar was included due to its atmospheric concentration. Fe is a known impurity in metakaolin and has some background contribution due to the stainless steel detector housing. Some known elements, such as O, were omitted because the X-ray fluorescence occurred at an energy below the threshold detected by the MAIA detector. Similarly, although Al and Si were included in the fit to the spectrum, their fluorescence occurred close to the low-energy cut-off of the MAIA detector. Consequently, sensitivity to Al and Si was relatively poor and quantitative analysis of their concentration in the sample was unreliable. Therefore, samples were later analysed by scanning electron microscopy energy-dispersive spectroscopy (SEM-EDS; Lyra3 TESCAN SEM) to map the distribution of Al and Si, together with O and Cs (see Section 4.4).

**Figure 5:**
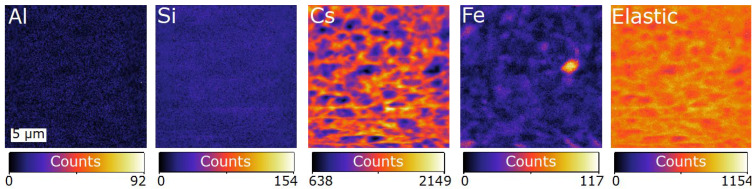
Maps of the signals fitted to the data obtained with the MAIA detector at the first time point (34 min after mixing). The sensitivity to Al and Si is low and produces maps with a low signal-to-noise-ratio, making it difficult to interpret distribution. The Cs map shows high contrast with inhomogeneous distribution, and can be correlated with the elastic scatter map, showing that it can be used to identify where the geopolymer has formed. Fe is a known contaminant in metakaolin.

Maps of the distribution of each element were obtained from the fitted spectra at each scan pixel, as shown in Figure 5. These images are shown using counts as there was significant uncertainty in the thickness and composition of the geopolymer paste at each time point, limiting the accuracy of quantification of the concentration. An highly anisotropic distribution of Cs is indicated by the SXFM images, strongly correlated with the elastic scatter in Figure 5, which further confirms that the Cs signal locates denser regions that contain the geopolymer. Due to the lack of sensitivity to Al and Si, the maps of their distribution in Figure 5 have a low signal-to-noise ratio, and therefore cannot be used to easily interpret their distribution, the distribution of metakaolin, or that of the geopolymerisation process.

**Figure 6:**
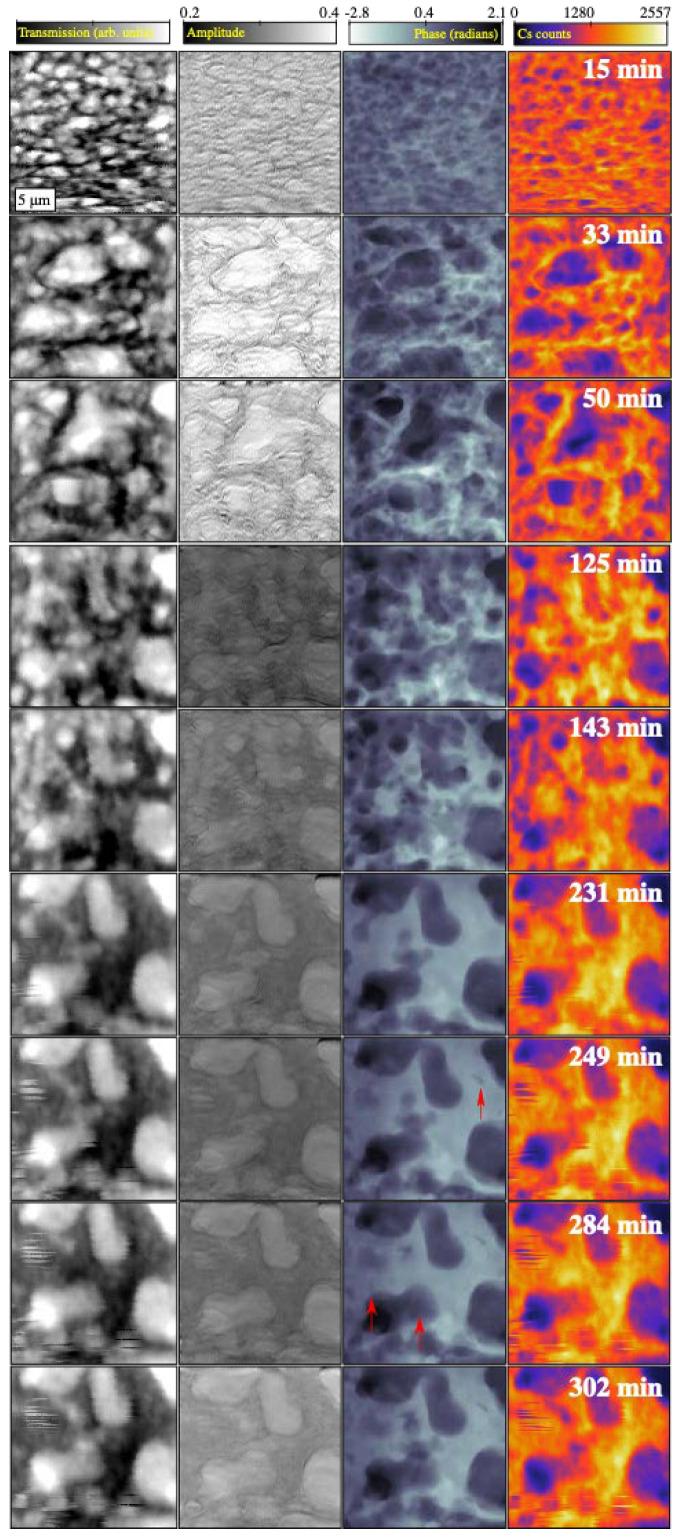
Comparison of simultaneously acquired scanning STXM images, SXDM amplitude and phase images, and the SXFM Cs distribution for selected intervals in the time-series data obtained from a geopolymer paste. These images were obtained over a continuous period immediately after mixing the metakaolin and caesium silicate and show the development of a porous geopolymer matrix incorporating caesium. Each image represents an area of 20 μm × 20 μm. Red arrows in the SXDM phase images indicate cracks that developed after the geopolymerisation process had slowed down.

The spatial resolution of SXFM is defined by the extent of the probe intensity at the object plane. The sum of the intensity of each probe mode simultaneously recovered during the reconstruction of the last SXDM dataset in the time series in Figure 6 is shown in Figure 3. The FWHM is 460 (H) × 751 (V) nm, which can be taken as the representative spatial resolution for all SXFM images.

### 4.3. Combinatorial Time-Series Images

The combination of fluorescence and imaging detectors used in this work allowed for the simultaneous acquisition of scanning X-ray diffraction microscopy (SXDM), scanning X-ray fluorescence microscopy (SXFM) and scanning transmission X-ray microscopy (STXM) images. A representative SXDM/SXFM time-series showing morphological changes in the product of the reaction between metakaolin and caesium silicate is shown in Figure 6. The phase images in Figure 6 represent a projection through the relatively thick sample and show a discontinuous network, with overlapping micron-scale pores that coalesce due to lateral redistribution of material as the reaction proceeds over approximately 4 h.

The precursors and products of the geopolymerisation process can be identified from the combination of SXFM and SXDM data. While the low sensitivity to light elements Al and Si in the SXFM images, noted in Section 4.2, limited the ability to directly distinguish some components, the structure and distribution of material containing predominantly Si and Al was clearly revealed by SXDM. This highlights the value of producing correlated SXDM and SXFM images.

No unreacted metakaolin is observed in the first phase image of the time-series (Figure 6), in contrast to the case shown in Figure 2b. It appears that the metakaolin was entirely dissolved during the 15 min it took to prepare the metakaolin/caesium silicate samples and locate regions of interest using the X-ray beam. A network of geopolymer phases with densely distributed sub-micron pores also formed in this time. This is remarkable considering that caesium silicate is known to slow geopolymerisation processes, in contrast to sodium silicate. The high initial water content was also expected to result in a slower dissolution process. However, water rapidly evaporated from the slurries deposited on to the silicon nitride membrane because of the high surface-to-volume ratio, which resulted in the pH increasing and the concomitant increase in the rate of metakaolin dissolution.

The highly anisotropic caesium distribution observed in all of the SXFM images in the time-series (Figure 6) indicates that a significant redistribution of caesium occurred as the geopolymerisation reaction progressed. High X-ray transmission in the SXTM images is strongly correlated with the regions of low caesium concentration indicated by the SXFM images, confirming the identification of pore features and that the caesium is mostly incorporated in the geopolymer at the first time point (*t* = 17 min). Caesium is also present within the pores, which are assumed to contain some liquid containing the caesium-based activator.

**Figure 7:**
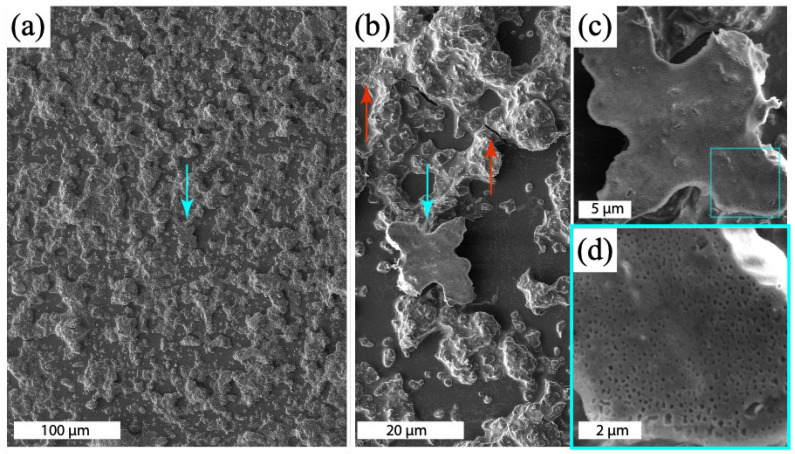
(**a**) SEM image of a cured geopolymer paste. The blue arrow highlights part of the geopolymer paste that dislodged from the silicon nitride substrate and flipped over, exposing a flat surface where sub-micrometre pores can be observed at the interface. Panels (**b**–**d**) show further details of the object and its pore structure. The red arrows in (**b**) indicate cracks in the geopolymer. Images were obtained using a Lyra3 TESCAN SEM with a 5 kV accelerating voltage and secondary electron detection.

Images obtained after 4 h show relatively insignificant changes in morphology, except for the appearance of nanoscale cracks. The features attributed to cracks are only resolved in the SXDM images and identifiable due to the strong phase contrast. The appearance of these features after approximately 4 h cannot be explained by the presence of unreacted metakaolin or development of pores. Cracks were also identified by scanning electron microscopy (SEM) performed on a similarly prepared geopolymer sample (Figure 7b).

### 4.4. SEM

SEM and energy-dispersive spectroscopy (EDS) data were collected on representative geopolymer samples 8 months after the experiment at the Australian Synchrotron was performed. SEM images of the cured geopolymer are shown in Figure 7. The observed morphology is consistent with that observed in the SXFM and SXDM images obtained after the reaction had proceeded for several hours in similarly prepared samples (Figure 6).

**Figure 8:**
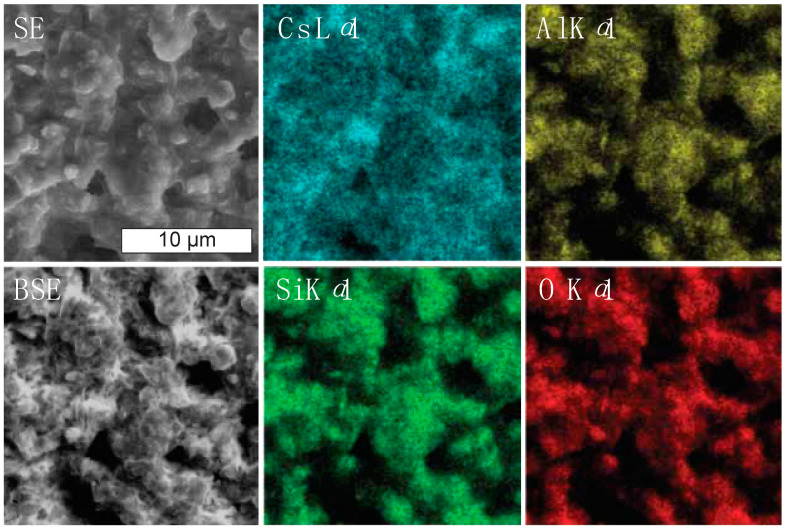
SEM micrographs and X-ray maps of a representative region of a geopolymer sample prepared similarly to the sample shown in Figure 6 and allowed to fully cure. The morphology observed by EDS-SEM is similar to the morphology observed after several hours by SXDM and was used to estimate the Al:Cs:Si ratio.

SEM-EDS analysis indicated the Al:Cs:Si ratio averaged over the cured samples was 1:1:1.5, which is close to the ratio in the target formulation, Cs_2_O·Al_2_O_3_·4SiO_2_·11H_2_O, of 1:1:2. The Cs distribution observed by SEM-EDS was less anisotropic than that observed by SXFM and weakly correlated with the distributions of O, Al and Si, which were strongly correlated with each other (Figure 8). Differences in the Cs distribution observed by SEM-EDS and SXFM may result from higher sensitivity in the former to residual Cs distributed across the geopolymer surface as the water evaporated from the solution.

## 5. Conclusions

The properties of geopolymers, their precursor materials, and the geopolymer formation process are all closely related to their compositional and physical heterogeneity on length scales of micrometres and below. We show that SXDM may be used together with SXFM to study the dissolution of precursor material and polycondensation processes in situ. Results demonstrate that the imaging sensitivity, material contrast and spatial resolution are sufficient for elucidating these processes in an extremely simple preparation of relatively thick samples. The temporal resolution was adequate to observe the nanoscale redistribution of material over a time scale of minutes.

The chosen liquid-to-solid ratio in addition to the high surface area resulted in fast metakaolin dissolution that was not temporally resolved. The rapid condensation process that followed led to the formation of material with high porosity that would, in practical applications, translate to low-strength bulk material. Further work is required to understand the influence of the size and morphology of the sample, and interaction of the sample in its liquid and gel phases with the substrate. In addition to providing higher X-ray contrast, caesium silicate is known to slow down the gel or geopolymer formation time by as much as five times when compared with Na- and K-based activators [37]. However, the gel formation time does not reveal the rate of dissolution of the precursor, which is also of critical importance in understanding the complete geopolymerisation process. The ability to distinguish metakaolin precursor from aluminosilicate gel phases by SXDM demonstrates the potential to quantify rates of both gel formation and the rate of precursor dissolution with the modest temporal resolution demonstrated in this work, if methods to control evaporation can be implemented. However, the simple method of depositing samples on X-ray transparent membranes successfully overcame the problem of the X-ray-beam-induced hydrolysis affecting the sample microstructure and allowed for the sufficient control of sample thickness. Therefore, a priority for future work lies in improving the temporal resolution and mitigating the influence of the X-ray beam on the microstructure by the optimisation and fractionation of the applied X-ray dose.

The recent availability of faster X-ray detectors [38] and SXDM optimisation has resulted in the potential for considerably faster data acquisition [38]. The development of algorithms to improve the reliability of reconstructing quantitative images [39,40] from time-series SXDM is expected to allow a significant improvement in temporal resolution, making the early stages of metakaolin dissolution and geopolymer condensation accessible.

The information obtained from this experiment will pave the way for future work where a range of different Si:Al and Cs:Al ratios are investigated with the aim of observing the extent of the reaction of metakaolin-based geopolymers in situ. Work by Williams et al. [41] revealed dramatic differences in the extent of the reaction of a metakaolin-based geopolymer after the setting and curing of samples. The ability to follow the dissolution, condensation, and initial setting dynamically with SXDM and SXFM will be a significant step forward in understanding the limitations in the existing production of geopolymers, where the extent of the reaction is not possible to predict in advance.

## Data Availability

The original contributions presented in the study are included in the article, further inquiries can be directed to the corresponding author.
